# Radiological and clinical outcomes after Attune primary total knee arthroplasty using Stemmable Tibia: A two-year follow-up prospective bi-center study

**DOI:** 10.1371/journal.pone.0309015

**Published:** 2024-08-29

**Authors:** Seung Joon Rhee, Seung Hun Woo, Jung Shin Kim, Mi Sook Yun, Chankue Park, Sang-Min Lee

**Affiliations:** 1 Department of Orthopedic Surgery, Biomedical Research Institute, Pusan National University Hospital, Busan, Korea; 2 Pusan National University, Pusan National University School of Medicine, Yangsan, Republic of Korea; 3 Department of Orthopedic Surgery, Research Institute for Convergence of Biomedical Science and Technology, Pusan National University Yangsan Hospital, Yangsan, Korea; 4 Division of Biostatistics, Research Institute for Convergence of Biomedical Science and Technology, Pusan National University Yangsan Hospital, Yangsan, Korea; 5 Department of Radiology, Research Institute for Convergence of Biomedical Science and Technology, Pusan National University Yangsan Hospital, Yangsan, Korea; Carol Davila University of Medicine and Pharmacy: Universitatea de Medicina si Farmacie Carol Davila din Bucuresti, ROMANIA

## Abstract

This prospective bi-center study aimed to analyze the outcomes of primary total knee arthroplasty using the Stemmable Tibia Attune system. A total of 100 patients who underwent primary total knee arthroplasty with Stemmable Tibia from January 2019 to December 2021 were enrolled in the study. Radiological outcomes (hip-knee-ankle axis and medial proximal tibial angle) were assessed preoperatively and postoperatively. Clinical outcomes (visual analog scale score, Hospital for Special Surgery score, Knee Society function score, Knee Society knee score, flexion contracture, further flexion, and range of motion) were analyzed preoperatively and at 6 weeks, 3 months, 6 months, 1 year, and 2 years postoperatively. Complications (periprosthetic joint infection and aseptic loosening) were examined. The hip-knee-ankle axis decreased (preoperative: 9.5° ± 6.3°, postoperative: 1.1° ± 2.7°), whereas the medial proximal tibial angle increased (preoperative: 84.6° ± 4.1°, postoperative: 89.8° ± 1.9°). The visual analog scale score, Hospital for Special Surgery score, Knee Society knee score, and Knee Society function score increased postoperatively. The Knee Society knee score indicated above good outcomes (100.0% and 99.0% at 1 and 2 years postoperatively, respectively). The Knee Society function score also showed above good results (98.0% and 93.0% at 1 and 2 years postoperatively, respectively). The range of motion significantly improved (p < 0.001): flexion contracture decreased from 9.10° ± 7.23° to 2.15° ± 2.87°, whereas further flexion increased from 136.05° ± 14.78° to 139.80° ± 10.02°. One patient developed periprosthetic joint infection; no early loosening was observed. In conclusion, Attune primary total knee arthroplasty with Stemmable Tibia not only is safe and effective but also leads to radiological and clinical improvements.

## Introduction

Total knee arthroplasty (TKA) is a well-established and cost-effective treatment for end-stage degenerative knee osteoarthritis [1–4]. Despite these advancements, the revision rate remains at 5% within 10 years postoperatively. The primary reasons for revision surgery are aseptic loosening (29.8%), infection (14.8%), and pain (9.5%) [5, 6].

The Attune TKA system (DePuy Synthes, Warsaw, IN, USA) was recently introduced, with the aim of enhancing patient outcomes and implant longevity [7]. However, Staats et al. reported a higher occurrence of radiolucent lines below the tibial component in medium-cemented TKA using Attune than that in its predecessor, P.F.C. Sigma (DePuy Synthes) [8]. The femoral and patellar components in the Attune TKA system have normal fixation; however, debonding frequently occurs between the implant-cement interface and the tibial component. These issues have been attributed to tibial surface roughness, polyethylene insert constraints, reduced cement pockets, and rotational stabilizers [9]. A biomechanical comparison study conducted by Jaeger et al. between the Attune TKA system and its predecessor and successor revealed issues with incomplete seating and tilting of the tibial component in the Attune TKA system (Attune and Attune S+) [7].

These challenges seem to be multifaceted and extend beyond design considerations [7–9]. The extension of the tibial implant in primary TKA enhances the stability of the implant [10, 11] by reducing micromotions at the bone-implant interface, promoting stability, and mitigating the risk of aseptic loosening, which is a main cause of revision. Nevertheless, no study has reported on the use of Stemmable Tibia in the Attune primary TKA system. Hence, this study aimed to analyze the short-term outcomes of primary TKA using the Stemmable Tibia (short-cemented stem of the Revision Knee System) in the Attune TKA system, evaluate physical functionality and patient-reported outcomes as components of clinical outcomes, assess implant survival up to 2 years postoperatively, and determine associated complications.

## Materials and methods

### Patients

**Fig 1:**
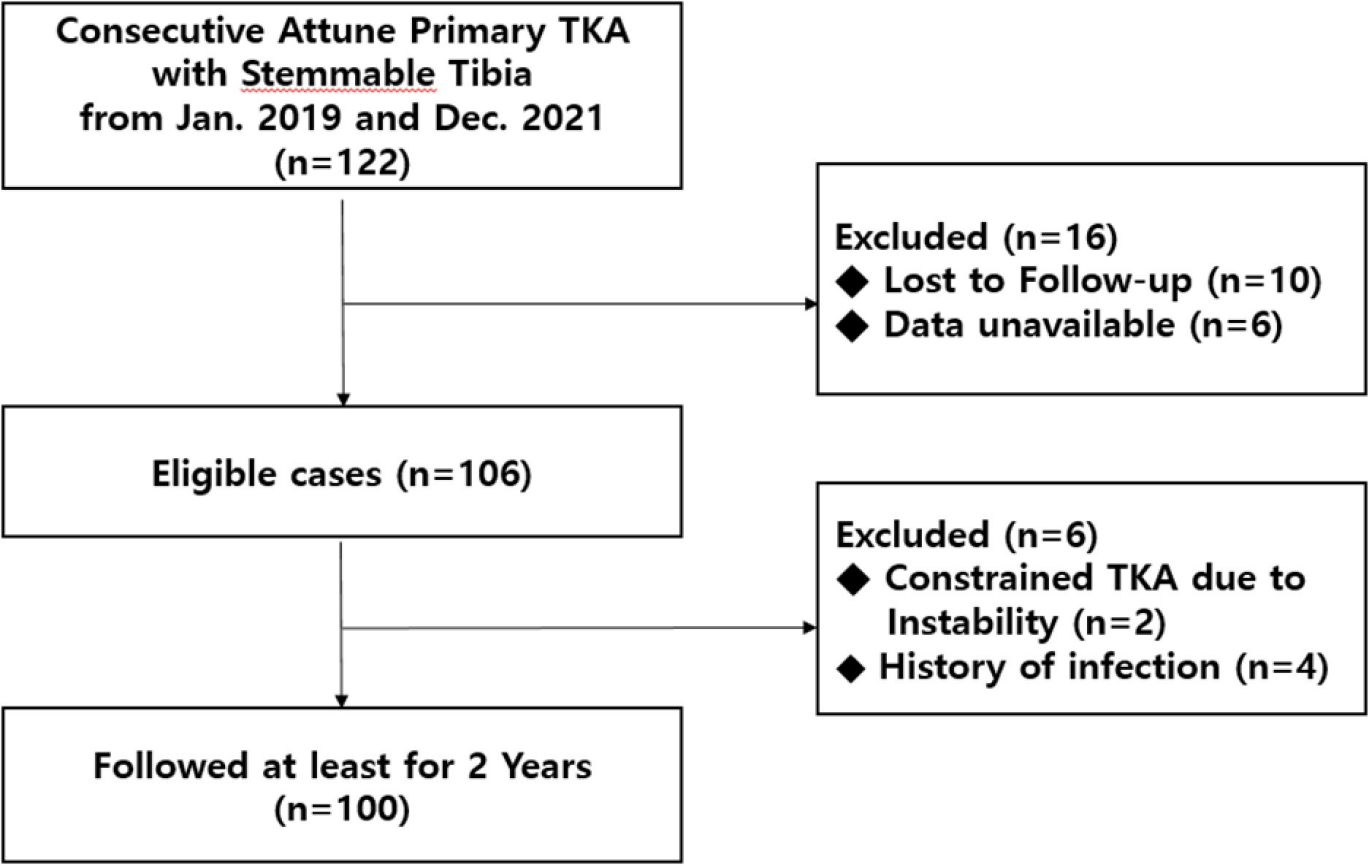
Flowchart of the data selection process in this study. TKA, total knee arthroplasty.

Data of all patients who underwent primary TKA from January 2019 to December 2021 were included in the study. All surgeries were performed at two hospitals by three surgeons using Attune primary TKA with Stemmable Tibia. According to the preformatted electronic database, 122 TKAs were performed within the study period. All data were accessed between January 2021 and December 2023. The inclusion criteria were old age, primary osteoarthritis diagnosis, rheumatoid arthritis, traumatic arthritis, or avascular necrosis unresponsive to conservative therapy. The minimum follow-up period was 2 years. Patients who were followed up for less than 2 years, those whose data were not available (participating in other surgical intervention or pain management studies), those who had past infection of the affected joint or history of local or systemic infection that could affect the prosthetic joint, and those who underwent constrained TKA due to instability were excluded (Fig 1). Preoperatively, sociodemographic data, including age, sex, height, weight, and body mass index (BMI), were collected using the electronic medical record of each patient.

### Surgical technique and postoperative care

All orthopedic surgeons (S.J.R., J.S.K., and S.-M.L.) participating in the surgical procedures received training on the DePuy Attune knee implant system and had prior experience with the system prior to the commencement of this study [12]. Each surgery involved the use of a single implant (Attune posterior stabilized knee implant with Stemmable Tibia) and followed the same technique [13]. Surgical planning relied on preoperative measurements from whole-leg radiographs, ensuring a vertically aligned postoperative mechanical axis. An anterior midline incision was made for bone cutting and insertion, followed by a medial parapatellar arthrotomy for the femoral component, resulting in a bone thickness equal to that of the femoral component. After patellar eversion, osteophytes were removed. The valgus angle of the femoral resection was determined using an intramedullary guide, and an anterior referencing system guided anteroposterior (AP) cutting. The femoral component size was measured during the femur operation based on the AP dimension of the femur.

After determining the femoral cutting guide size, the rotation of the femoral component was adjusted based on the preoperatively measured transepicondylar axis and gap. Subsequently, femoral bone cutting was performed using the femoral cutting guide. The positioning of the femoral component cutting guide was set at 2° or 7° of external rotation to the posterior condyles, as verified using the AP trochlear sulcus.

Minimal tibial bone resection was performed to obtain a surface perpendicular to the tibial shaft in the coronal plane. The reference line for tibial rotation was accurately aimed at a line passing through the medial third of the tibial tubercle and the second metatarsal or middle of the talus [14]. All osteophytes were removed. After the femoral and tibial bones were cut, a trial component was inserted. If necessary, soft tissue release was performed to equalize the gap balance [13]. Patellar tracking was assessed for each trial component using the no-thumb test. All TKAs were planned to use a posterior-stabilized prosthesis.

Postoperatively, all patients were treated using a standardized rehabilitation protocol, including pain management through patient-controlled analgesia and oral narcotics, removal of compression dressings, and closed suction drain after 24 h. Quadriceps muscle exercise was started on the first postoperative day, followed by continuous passive motion and physical therapy on the second postoperative day, to facilitate protected ambulation, in addition to range of motion (ROM) exercises. Deep venous thrombosis prophylaxis with enoxaparin was commenced on the second postoperative day, and the patients were discharged from the hospital at 14 days postoperatively [13].

### Radiological assessment

All images were obtained by a skilled technician according to standard protocols and included preoperative and 1-year postoperative knee radiographs obtained at different views, including standing the AP view, lateral view, weight-bearing posteroanterior view at 45° of flexion, and full-length standing AP view.

The following measurements were obtained from preoperative and 1-year postoperative full-length standing AP radiographs: hip-knee-ankle (HKA) axis, lateral distal femur angle (LDFA), and medial proximal tibial angle (MPTA). The HKA axis denoted the angle of the femoral and tibial mechanical axes, with varus alignment designated as positive. The MPTA was defined as the medial angle between the tibial mechanical axis and the proximal tibial joint line (the connection of the lowest points of the medial and lateral tibial plateau), whereas the LDFA represented the lateral angle between the femoral mechanical axis and the distal femoral joint line (the connection of the lowest points of the medial and lateral femoral condyle) [15]. Additionally, the HKA axis, coronal femoral component angle (CFA), and coronal tibial component angle (CTA) were measured on postoperative images. The mechanical leg axis was initially defined as the HKA angle. This angle represented the intersection between two lines: one connecting the center of the hip to the center of the knee, essentially depicting the mechanical axis of the femur, and the other linking the center of the knee to the center of the ankle joint, thereby characterizing the mechanical axis of the tibia. CFA was determined by calculating the inner angle between the mechanical axis of the femur and a horizontal reference line extending across the two prosthetic condyles. Similarly, CTA was ascertained by measuring the medial angle between the mechanical axis of the tibia and the horizontal axis of the tibial tray [16]. Furthermore, preoperative and postoperative LDFA and MPTA were taken as parallel to the CFA and CTA, and the differences were compared.

At both centers, radiological measurements were performed using a digital caliper from the Picture Archiving Communication System M6 (INFNITT Health Care, Seoul, Republic of Korea). An identical digital goniometer was used for all the evaluations. All radiological data were analyzed by two independent orthopedic surgeons (S.J.R. and S.-M.L.) with more than 10 years of experience.

### Clinical assessment

During the study period (preoperatively and at 6 weeks, 6 months, 1 year, and 2 years postoperatively), the following questionnaires were administered: visual analog scale (VAS) [17, 18], Knee Society function score (KS-FS), and Knee Society knee score (KS-KS) [19]. Flexion contracture (FC), further flexion (FF), and ROM [18] were measured using the goniometer and through physical examinations by an orthopedic surgeon, whereas the Hospital for Special Surgery (HSS) score [20] was assessed through interviews. The measured values were compared at the different time points. KS-KS ≥90, ≥77, ≥65, and <65 indicated excellent, good, fair, and poor scores, respectively, according to Miralles-Muñoz et al. [21], whereas KS-FS was categorized as ≥85, ≥73, ≥56, and <56. The proportion of patients with improvements above the cutoff value in each time frame was analyzed.

### Complications and survival analysis

Complications were classified as major (periprosthetic joint infection [PJI], aseptic loosening, and periprosthetic fracture or bone breach) or minor (need for subsequent procedures and wound dehiscence) [14, 22–27]. Survival was assessed based on the survival rate, assuming that patients who underwent revision surgery owing to a major complication were excluded during the yearly follow-up [28].

### Statistical analysis

Statistical analysis was performed using IBM SPSS Statistics version 28.0 (IBM Corp., Armonk, NY, USA). Significant differences in radiological variables were compared preoperatively and postoperatively using the paired t-test or Wilcoxon signed-rank test. For clinical assessment outcomes at various time points, repeated-measures analysis of variance was used. Uncensored and cumulative survival rates were estimated using the Kaplan-Meier survival analysis. The intraclass correlation coefficient (ICC) was used to evaluate the reliability of radiological measurements. Intrarater and interrater reliabilities were evaluated using the ICC. An ICC of 1 indicated perfect reliability, whereas an ICC of 0 indicated unreliability. The interrater ICC scores for preoperative alignment, LDFA, JLCA, and MPTA were 0.865, 0.864, 0.835, and 0.820, respectively, and the intrarater ICC scores were 0.890, 0.915, 0.846, and 0.855, respectively. The interrater ICC values for the postoperative alignment, LDFA, and MPTA were 0.867, 0.831, and 0.896, respectively, and the intrarater ICC values were 0.872, 0.870, and 0.901, respectively, indicating reliable measurements. A p-value <0.05 was considered statistically significant.

### Ethical considerations

Medical data were reviewed by the Human Subjects Committee of Pusan National University. All study protocols were approved by the institutional review board (IRB no. 05-2020-259). Informed consent was obtained from all patients.

## Results

Of the 122 patients, 22 were excluded (follow-up <2 years, 10 patients; unavailable data, 6 patients; with previous infection of the affected joint or with local or systemic infection, 4 patients; and underwent constrained TKA due to instability, 2 patients). Finally, 100 patients who underwent primary TKA with Stemmable Tibia were included. The patient demographics are presented in Table 1.

**Table 1:** Patient demographics.

Parameters	Patient data	
All cases	100	
Sex (%)		
Male	28 (28)	
Female	72 (72)	
Age (years)	71.8 ± 5.4 [57–82]	
BMI (kg/m^2^)	26.8 ± 5.2 [14.6–36.9]	
Weight (kg)	63.5 ± 10.5 [41.0–86.6]	
Height (cm)	154.5 ± 7.8 [135.9–178.8]	
Side		
Right		50 (50)
Left		50 (50)
Follow-up duration (months)	31.9 ± 4.49 [24–38]	

Data are presented as n/n, number (proportion, %) or mean ± standard deviation [range].

BMI, body mass index.

### Radiological outcomes

The average preoperative scores for the HKA axis, LDFA, and MPTA were 9.45° ± 6.31°, 90.52° ± 3.53°, and 84.55 ± 4.06°, respectively. The average postoperative scores for the HKA axis, CFA, and CTA were 1.13° ± 2.65°, 90.69° ± 1.08°, and 89.83° ± 1.92°, respectively. Changes in the HKA axis and MPTA before and after surgery were significant (p < 0.001) (Table 2).

**Table 2:** Radiological outcomes.

Parameters	Preoperative (°)	Postoperative (°)	p-value
HKA axis	9.45 ± 6.31	1.13 ± 2.65	<0.001
LDFA (CFA)	90.52 ± 3.53	90.69 ± 1.08	0.644
MPTA (CTA)	84.55 ± 4.06	89.83 ± 1.92	<0.001

Data are presented mean ± standard deviation.

HKA, hip-knee-ankle; LDFA, lateral distal femur angle; MPTA, medial proximal tibial angle; CFA, coronal femoral component angle; CTA, coronal tibial component angle.

### Clinical outcomes

**Fig 2:**
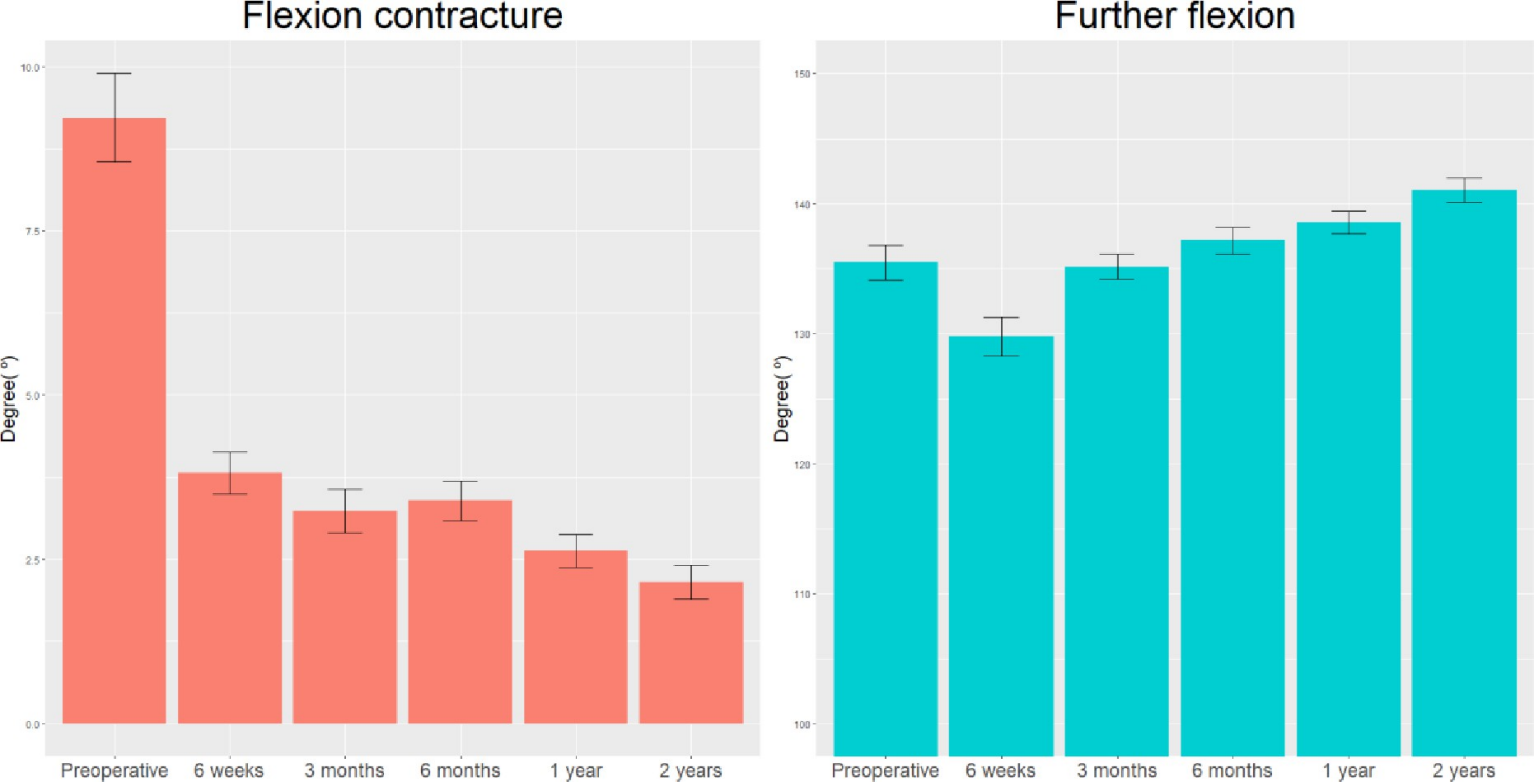
Changes in flexion contracture and further flexion over time.

The preoperative mean and final mean FC were 9.10° ± 7.23° and 2.15° ± 2.87°, respectively. The preoperative mean FF was 136.05° ± 14.78°, which significantly increased to 139.80° ± 10.02° at 2 years postoperatively (p < 0.001) (Fig 2). Consequently, ROM also significantly increased from 126.95° ± 15.67° to 137.65° ± 10.41° (Table 3).

**Table 3:** Range of motion.

	Time point							p-value	
	Preoperative	6 weeks	3 months	6 months	1 year	2 years	vs. preoperative		Overall
**FC**	9.10 ± 7.23	4.15 ± 4.08	3.40 ± 4.08	2.75 ± 3.36	2.35 ± 2.97	2.15 ± 2.87	<0.001		<0.001
**FF**	136.05 ± 14.78	129.90 ± 15.06	134.65 ± 11.26	134.40 ± 9.37	138.65 ± 8.16	139.80 ± 10.02	<0.001		<0.001
**ROM**	126.95 ± 15.67	125.75 ± 14.74	131.25 ± 12.19	131.65 ± 9.90	136.30 ± 8.84	137.65 ± 10.41	<0.001		<0.001

Data are presented mean ± standard deviation.

FC, flexion contracture; FF, further flexion; ROM, range of motion.

With respect to the VAS score, a significant decrease was observed from the preoperative average of 6.90 ± 1.55 to the final follow-up average of 1.11 ± 0.72 (p < 0.001). Similarly, the HSS score significantly increased from the preoperative average of 54.47 ± 6.01 to the final follow-up average of 92.39 ± 6.44 (p < 0.001). A significant difference in the KS-KS was observed between the preoperative score of 42.71 ± 7.41 and the final score of 89.89 ± 4.54 (p < 0.001). Likewise, a significant difference in the KS-FS was found between the preoperative score of 51.78 ± 3.70 and the final score of 82.57 ± 6.62 (p < 0.001) (Table 4).

**Table 4:** Clinical outcomes.

	Time point	Measured value	p-value	
			vs. Preoperative	Overall
VAS	Preoperative	6.90 ± 1.55	-	<0.001
	6 weeks	5.13 ± 1.81	<0.001	
	3 months	4.42 ± 1.45	<0.001	
	6 months	2.75 ± 1.19	<0.001	
	1 year	1.21 ± 0.82	<0.001	
	2 years	1.11 ± 0.72	<0.001	
HSS	Preoperative	54.47 ± 6.01	-	<0.001
	6 weeks	59.41 ± 4.49	<0.001	
	3 months	77.38 ± 7.27	<0.001	
	6 months	84.96 ± 6.25	<0.001	
	1 year	90.98 ± 5.51	<0.001	
	2 years	92.39 ± 6.44	<0.001	
KS-KS	Preoperative	42.71 ± 7.41	-	<0.001
	6 weeks	67.94 ± 10.18	<0.001	
	3 months	78.24 ± 7.93	<0.001	
	6 months	87.24 ± 6.16	<0.001	
	1 year	91.11 ± 3.34	<0.001	
	2 years	89.89 ± 4.54	<0.001	
KS-FS	Preoperative	51.78 ± 3.70	-	<0.001
	6 weeks	54.89 ± 5.58	<0.001	
	3 months	64.67 ± 3.86	<0.001	
	6 months	73.07 ± 5.43	<0.001	
	1 year	82.08 ± 5.03	<0.001	
	2 years	82.57 ± 6.62	<0.001	

Data are presented mean ± standard deviation.

KS-FS, Knee Society function score; HSS, Hospital for Special Surgery; KS-KS, Knee Society knee score; VAS, visual analog scale.

The KS-KS was graded above good: 100.0% at 1 year postoperatively and 99.0% at 2 years postoperatively. The KS-FS was graded above good: 98.0% at 1 year postoperatively and 93.0% at 2 years postoperatively. Both indicators significantly varied postoperatively, as compared with those preoperatively (p < 0.001) (Table 5).

**Table 5:** A validated outcome categorization of the Knee Society score.

Time point	KS-KS					KS-FS				
	Poor	Fair	Good	Excellent	p-value	Poor	Fair	Good	Excellent	p-value
**Preoperative**	98.0	2.0			<0.001	88.0	12.0			<0.001
**6 weeks**	37.0	41.0	20.0	2.0		59.0	41.0	-	-	
**3 months**	4.0	37.0	53.0	6.0		-	99.0	1.0	-	
**6 months**	-	6.0	53.0	41.0		-	47.0	51.0	2.0	
**1 year**	-	-	32.0	68.0		-	2.0	67.0	31.0	
**2 years**	-	1.0	43.0	56.0		-	7.0	51.0	42.0	

Data are presented as percentages (%).

Dashes (-) indicate non-applicable areas.

KS-FS, Knee Society function score; KS-KS, Knee Society knee score.

### Complications and survival analysis outcomes

**Fig 3:**
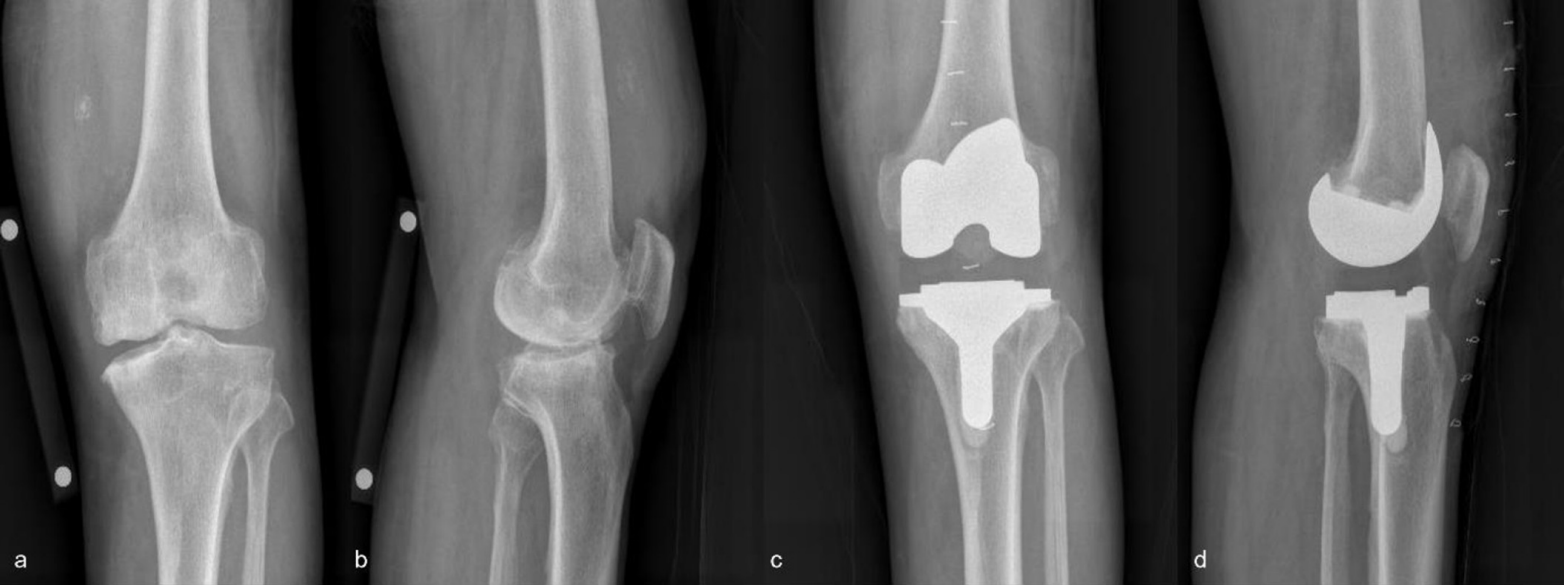
(a and b) Advanced osteoarthritis in a 67-year-old patient and (c and d) total knee replacement performed on the patient. A tibial posterior cortical bone breach is observed on postoperative radiography. No pain or signs of progression were observed on follow-up. The decision was made to not proceed with revision surgery.

Postoperative complications included two major cases of PJI and posterior cortical breach during stem reaming and two minor cases of wound dehiscence and stiffness during the brisement maneuver. PJI occurred at 8 months postoperatively, for which prosthesis with antibiotic-loaded acrylic cement (PROSTALAC) and revision TKA were performed. Conservative therapy was applied to the posterior cortical bone breaches during tibial stem reaming (Fig 3). No subsequent complications occurred, and no loosening was observed. Accordingly, the survival estimate was 99% at 2 years postoperatively.

## Discussion

This study demonstrated the positive outcomes of using Stemmable Tibia in the Attune primary TKA system. Radiological results showed that the HKA axis improved, with noteworthy changes in the MPTA. Clinically, FC decreased, whereas FF and ROM increased. Additionally, the VAS score decreased, whereas the HSS score, KS-KS, and KS-FS significantly increased.

A previous study reported an unusually high rate of early failure of the Attune tibial component for a novel TKA design. Early loosening was attributed to reduced stability at the tibial implant-cement interface, primarily caused by reduced rotational stabilizers and stem length due to reduced tibial plate cement pockets and roughness factor [9]. Jaeger et al. also noted issues with tibial component fit and instrumentation, leading to incomplete seating in the Attune system [7]. Compared with the previous design, incomplete contact on the bone surface increased for the Attune tibial component unless an additional force was applied. This was attributed to the rounded edges with a 3-mm radius at larger radii among the keel, stem, and tibial plateau base in the Attune system compared with its predecessors. Further, the lack of space created by the tibial instruments and keel punches to fit the curved design features caused a press fit and increased seating resistance. Excessive press fit and uneven bone quality or sclerosis leads to tilting of the tibial component during cementation or cement polymerization, resulting in incomplete seating [7]. As such, Stemmable Tibia can potentially improve debonding resulting from recessed or surface textured cement pockets for cement fixation [29]. Thus, we applied Stemmable Tibia and did not observe early loosening, except for an infection; no revision surgery was required, either. Additionally, clinical outcomes were satisfactory (>90% of patients had an excellent or good level of satisfaction). Nevertheless, further studies should investigate cases with radiolucent lines.

A study comparing the propensity score matching between the short- and non-stem groups (n = 602 patients), with adjustments for age, sex, BMI, preoperative mechanical axis, and postoperative alignment, reported a decline in the rate of loosening in the presence of varus deformity [30]. Similarly, Garceau et al. reported a decrease in the aseptic loosening frequency of the short stem in primary TKA [31]. Stemmable Tibia is similar to a short-stem tibia with a total height of 46 mm in the tibial component, which is higher than the maximal height of 43 mm for Attune primary tibia; hence, a higher level of stability is likely achieved. Moreover, the length did not exceed the nonextended stem at 50 mm in the primary tibial component of a different manufacturer, for verification of its safe use in the primary tibia [32].

The Norwegian Arthroplasty Register reported an increase in 10-year survival to 94%, from 2005 to 2015 [33], and the Swedish Knee Arthroplasty Register reported an increase in 10-year survival to 89%, from 1985 to 1994, and 96%, from 2005 to 2014 [14]. In this study, one patient received a PROSTALAC and underwent revision surgery due to PJI, and the survival estimate at the 2-year follow-up was 0.99. Despite this high survival estimate, a longer follow-up period is needed to draw a conclusion for Stemmable Tibia.

This study had some limitations. First, the sample size was small; therefore, it is difficult to attribute general clinical significance to the study results. However, this is meaningful because it was a prospective and bi-center study. Second, this was an observational cohort study, and comparison with other prostheses was difficult. Third, although the results of this study are promising, the number of patients with a 2-year follow-up period was relatively low. Longer follow-up periods and further studies are needed to determine the long-term survival and clinical performance of Stemmable Tibia implants.

In conclusion, the results of this study on Attune primary TKA with Stemmable Tibia showed significant changes in the HKA axis and MPTA (CTA) as radiological outcomes; no findings indicated aseptic loosening. Significant improvements in clinical outcomes (ROM, VAS score, HSS score, KS-KS, and KS-FS) were detected, indicating that the method was safe and effective. However, further research, including long-term studies, is required.

## Supporting information

S1 Data (XLSX)
